# Vector Acceleration Methods for Faster Convergence of Cyclic Steady State in Adsorption Process Simulations

**DOI:** 10.1021/acs.iecr.6c00407

**Published:** 2026-04-28

**Authors:** Sai Gokul Subraveti, Kian Karimi, Matteo Gazzani

**Affiliations:** † SINTEF Energy Research, Sem Sælands vei 11, Trondheim 7034, Norway; ‡ Copernicus Institute of Sustainable Development, Utrecht University, Princetonlaan 8a, Utrecht 3584, CB, The Netherlands; § Sustainable Process Engineering, Chemical Engineering and Chemistry, Eindhoven University of Technology, Eindhoven 5612, AP, The Netherlands

## Abstract

Simulations of fixed-bed processes with in situ adsorbent regeneration, which are particularly important for adsorption-based separations, require the repeated solution of nonlinear partial differential equations over multiple cycles, starting from predefined initial conditions and continuing until a cyclic steady state (CSS) is reached. This iterative approach, typically based on successive substitution, poses a significant computational challenge, making simulation and optimization time-consuming. The present study systematically investigated several variants of Steffensen’s vector acceleration methods for their ability to expedite CSS convergence in adsorption process simulations. These methods are straightforward to implement, requiring no prior knowledge of the first derivatives (or Jacobian). Three different adsorption processes, each with unique cycle dynamics, are considered to be test cases: a four-step vacuum swing adsorption (VSA) cycle, a six-step temperature swing adsorption (TSA) cycle, both designed for postcombustion CO_2_ capture, and a three-step vacuum temperature swing adsorption (VTSA) cycle designed for CH_4_ upgrading from atmospheric sources. The results show that these methods, particularly the Graves-Morris extrapolator, can consistently enhance the rate of CSS convergence across the different applications. More specifically, in VSA simulations, the computational times are reduced to a quarter of that needed by successive substitution. In TSA and VTSA simulations, the methods reduced times by up to 70 and 35%, respectively. The analysis demonstrated the potential for accelerating CSS convergence through simple, code-agnostic modifications.

## Graphical abstract

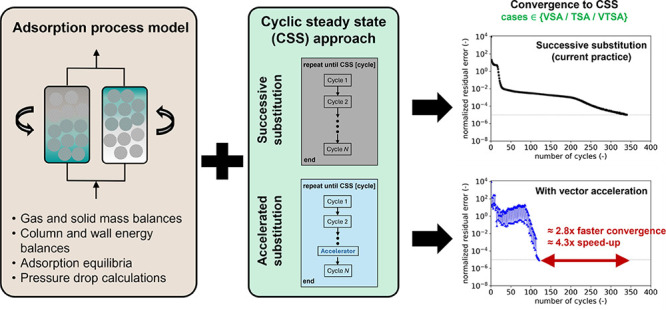

## 1 Introduction

Adsorption processes are widely used for various industrial gas separations.^1,2^ These processes typically employ one or more fixed-bed adsorption columns packed with a selective adsorbent material and operate cyclically, alternating between adsorption and desorption steps to achieve the desired separation. Depending on the chosen desorption (regeneration) method, which can be achieved by varying the pressure, temperature, concentration, or a combination of these variables, different process configurations can be designed. The most common include pressure swing adsorption (PSA), temperature swing adsorption (TSA), vacuum swing adsorption (VSA), and vacuum temperature swing adsorption (VTSA).^3^

While conventional separation methods, such as distillation and absorption, operate under steady-state conditions, adsorption processes are transient and modular, operating under a cyclic steady-state (CSS) condition rather than a true steady state.^2^ From a process design standpoint, adsorption columns exhibit both spatial and temporal variations and are mathematically represented by a set of nonlinear partial differential equations (PDEs) resulting from mass, momentum, and energy balances.^4^ Starting with an arbitrary initial condition, these equations are solved step by step for each cycle sequentially, with the solutions being fed into the next cycle. The calculations are repeated over multiple cycles until the system reaches CSS. At CSS, these profiles no longer vary as the numerical iterations continue, and the key process performance indicators are then calculated based on the CSS transient profiles of state variables. This iterative approach, commonly referred to as *successive substitution* in adsorption process literature, follows *Picard* or *fixed-point iteration* (FPI). To maintain consistency throughout this article, we refer to this method as the *successive substitution* approach.

Notably, adsorption processes are modular, with several constituent steps in each cycle. The selection and sequencing of steps, together with the direction of flows, can give rise to multiple cycle configurations (distinct processes) that may perform substantially differently from one another. This, along with the nonintuitive selection of several steps timing, requires rigorous mathematical optimization of the process.^5–7^ However, optimizing these processes remains computationally demanding; while several contributing factors exist,^8^ the repeated solution of nonlinear PDEs required to reach CSS remains the most challenging aspect. Although a single simulation may take only minutes on one core,^9^ optimization across thousands of operating conditions and for a multitude of adsorbents^10^ demands millions of core hours, even with parallel computing.^3^ This computational problem is further exacerbated by the ability to synthesize different processes for each of these adsorbents and optimize each adsorbent-process combination. In this context, data-driven models have recently been developed and adopted as surrogates for physics-based models, particularly to enable rapid evaluations in integrated adsorbent-process optimizations.^11,12^ However, these models still depend on the availability of large training data sets generated from the physics-based models. Hence, accelerating the convergence of CSS in adsorption process simulations remains a critical task, offering benefits to both physics-based modeling and machine learning frameworks.

Efforts to accelerate convergence to CSS date back to the early 1990s,^13^ with suggested acceleration methods broadly falling into three categories. The first involves shooting methods, which reformulate the CSS determination as a nonlinear root-finding problem. In their early works, Croft and LeVan^14^ applied Newton’s method to achieve quadratic convergence near the solution, reducing the number of cycle iterations from 30,000 to 15 for a nonlinear adsorption system. However, computing the Jacobian each cycle increased the computational time significantly.^15^ To avoid such extra computational overheads, quasi-Newton methods are employed in which the exact Jacobian is calculated in the initial iteration and then updated using multidimensional secant approximations. Smith and Westerberg,^16^ as well as Kvamsdal and Hertzberg,^15^ used Broyden’s method to update a mainly diagonal Jacobian for their linear adsorption systems. However, in strongly nonlinear systems, such as gas adsorption processes, these methods may struggle to achieve the desired convergence rates as the underlying assumption of the diagonal Jacobian becomes invalid.^17^ The subsequent paper from Ding and LeVan^17^ proposed a hybrid Newton-Broyden method combined with sensitivity interpolation and dynamic grid allocation to reduce Jacobian computations by calculating the selected Jacobian elements and interpolating the remaining ones. The success of this method relies on choosing the right number of interpolation elements, which can be challenging due to varied and complex nonlinear behaviors introduced by novel adsorbents.

In the second category, Wilson et al.^18^ applied perturbation techniques, including zeroth-order multiple-scale analysis and the Krylov-Bogoliubov (K-B) averaging, to speed up CSS convergence in oxygen VSA simulations. By decomposing the adsorbent temperature into fast (adsorptive) and slow (convective) time scales, this approach provided quick, approximate CSS temperature profiles under certain process conditions. However, ignoring the convective term on short time scales reduced the accuracy. Although first-order solutions are introduced to improve accuracy, excluding the mass balance from the analysis limits their applicability.

The third category, accelerated successive substitution methods, aims to speed up successive substitution (or Picard iterations) through various strategies. One such approach^19^ involves dynamically switching between simple and detailed mass transfer models, where the latter is used only near the CSS solution. Alternatively, Todd et al.^20^ employed adaptive node refinement for the spatial discretization of the PDEs, where simulations start with a coarse discretization and refine as the CSS is approached. Recently, Pai et al.^12^ trained artificial neural networks (ANNs) on hundreds of CSS profiles to provide better initial guesses for four-step VSA cycle simulations, enabling faster convergence to CSS. Another approach uses derivative-free extrapolation methods to estimate a new guess based on initial conditions from previous cycles. For instance, Kvamsdal and Hertzberg^15^ compared Aitken’s Δ^2^ method^21^ and Muller’s method for accelerating convergence in linear trace systems. In contrast, Choong et al.^22^ applied extrapolators only in the quasi-linear convergence region, where these techniques are most effective. Friedrich et al.^23^ achieved up to 10× faster convergence in dual piston PSA simulations by combining node refinement, ϵ extrapolation algorithm,^24^ and restart from previous simulations, while each strategy alone gave around 2× speed-up. More generally, from an implementation perspective, FPIs through successive substitution over multiple cycles produce a vector sequence of state variables. Under certain conditions, this sequence can be transformed into a new sequence using vector acceleration methods that converge faster to the same limit,^25^ without requiring the Jacobian or its inverse, making them simpler to implement. Because they rely on the principle of extrapolation, they are also termed derivative-free extrapolation methods.

Although various strategies have been proposed to accelerate CSS convergence, no single approach has yet to gain universal consensus,^18^ indicating the need for further investigation.

In this study, we build upon and extend our earlier work^26^ to thoroughly examine the use of vector acceleration methods, particularly derived from Steffensen’s method,^27^ for expediting CSS convergence in adsorption process simulations. The main motivation and contributions of this paper are summarized below:

- Kvamsdal and Hertzberg^15^ initially applied Aitken’s Δ^2^ method to accelerate the convergence of CSS. Over time, several vector extensions of this approach have emerged, making this an active research topic.^28,29^ As no universal acceleration method exists for all types of sequences,^28^ this paper presents a comprehensive investigation of 12 vectorized variants of Steffensen’s methods and evaluates their ability to enhance CSS convergence in adsorption process simulations. Among them is the ϵ_2_ algorithm, a special case of ϵ method used by Friedrich et al.^23^
- While Aitken’s Δ^2^ method was employed for linear trace systems,^15^ its effect on the CSS convergence in strongly nonlinear adsorption systems is unclear.
- This study tests the acceleration methods for three different adsorption processes, each with unique cycle dynamics, namely, a four-step vacuum swing adsorption cycle, a six-step temperature swing adsorption cycle (both for postcombustion CO_2_ capture), and a three-step vacuum temperature swing adsorption cycle for CH_4_ upgrading.
- We test the acceleration methods using two different adsorption process modeling frameworks—one implemented in Python and the other in Fortran—demonstrating that the acceleration remains effective across different implementation platforms and solvers.
- Finally, this work evaluates the acceleration methods across multiple simulations covering the entire design space to understand their true impact and analyzes how acceleration parameters affect oscillations or unstable initial guesses in the convergence of CSS.

## 2 Process Simulation

### 2.1 Mathematical Model and Numerical Approach

As briefly discussed in the Introduction, the dynamics of adsorption columns can, in most cases, be adequately described by a one-dimensional mathematical model comprising a set of coupled nonlinear PDEs.^9^ For brevity, the model equations are provided in the Supporting Information. The following key assumptions are made to derive these equations from the underlying laws of mass, energy, and momentum conservation: (1) gas-phase behavior is ideal; (2) bulk flow is characterized by axially dispersed plug flow; (3) the momentum balance is steady-state, and the pressure drop is calculated using the Ergun equation; (4) the linear driving force (LDF) model governs mass transfer from the bulk gas phase to the solid; (5) state variables such as composition, pressure, and temperature have no radial gradients; (6) thermal equilibrium is instantaneous between the gas and solid phases; and (7) uniform column properties.

Adsorption process models are solved numerically by adopting any adequate strategy, which typically relies on discretizing the continuous equations, i.e., by using finite differences, finite elements, or finite volumes. In this context, the method of lines, where PDEs are discretized in space while leaving time continuous, has proven to be particularly successful. An example of application to adsorption processes is provided by Haghpanah et al.;^9^ in the process models used for this work, we adopt a similar numerical strategy, which is implemented in Python 3.13 and Fortran 2023. First, the spatial terms in the PDEs are discretized into finite volumes by using a total variation diminishing (TVD) scheme with the van Leer flux limiter. The resulting system of stiff ordinary differential equations is integrated using ODE solvers: “*solveivp*” in Python 3.13, which employs the implicit multistep variable order scheme,^30^ and “*LSODE*” in Fortran 2023.^31^ The process simulations are carried out by assuming a unibed approach, where a single column undergoes all constituent steps in a sequence. This approach significantly reduces the excessive computational burden of simulating multiple columns at every time step. The column is initially assigned an arbitrary initial condition, typically assuming saturation with either the feed gas or one of the components of the gaseous mixture. Based on this initialization, the constituent steps are simulated sequentially.

### 2.2 Successive Substitution

The process simulations involve simulating several cycles (or cycle iterations) that progress from the predefined initial state to the CSS condition through successive substitution, as schematically represented in Figure 1. Once the CSS condition is established, key process performance metrics are obtained from the resulting CSS solution. If a black-box function, F , represents the adsorption process simulator, then the successive substitution (or FPI) can be expressed as,

$$\Delta_{n+1}=F\left(\Delta_{n}\right)\;\;\;\;\Delta \in R^{N},F:R^{N}\to R^{N}$$ 1

In eq 1, the adsorption process simulator, F , takes the final state of the adsorption column (in the last constituent step) from the *N*th cycle (Δ_*n*_) as an input and returns the final state of the adsorption column for the (*N* + 1)­th cycle. Note that Δ represents a vector of state variables, such as gas-phase composition, adsorbed amount, pressure, and column and wall temperatures across the adsorption columns.

**Figure 1:**
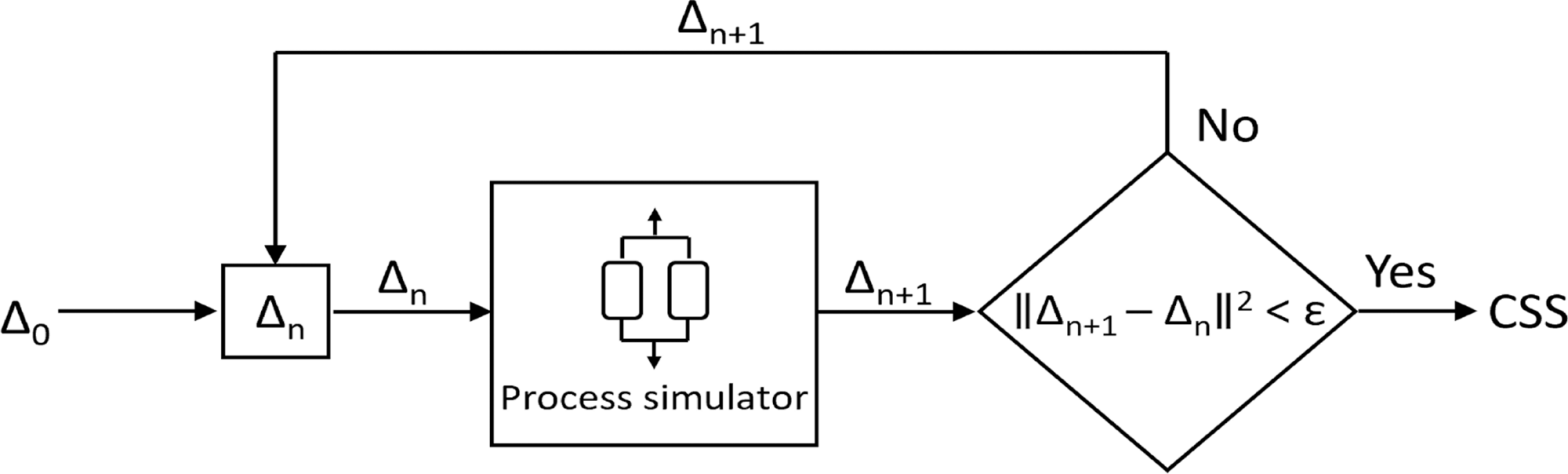
Schematic of an adsorption process simulator using successive substitution. Adapted from Effendy et al.^32^

Convergence to CSS, especially in bulk separations, often requires several cycle iterations, ranging from a few dozen to a few thousand depending on system nonlinearity and overall cycle times. Multiple studies have confirmed that, near the CSS solution, many PSA and TSA processes exhibit slow and linear convergence,^14,22,32^ which could be attributed to possessing a dominant Floquet multiplier less than unity (see Croft and Levan,^14^ Effendy et al.^32^). While successive substitution remains the most robust, straightforward method for solving nonlinear systems without requiring prior knowledge of the function F or its derivative, its inherently linear and slow convergence makes it computationally inefficient.

### 2.3 Cyclic Steady State

Several mathematical criteria have been employed to define CSS in adsorption process simulations.^32^ Few studies have assumed that CCS is reached after a large number of cycle iterations,^33,34^ while others define it based on when the absolute differences in axial and temporal profiles of state variables^7,9^ or absolute overall mass balance errors^6^ in consecutive cycles fall below a specified tolerance. Moreover, Rao et al.^35^ defined CSS as the condition in which the change in product purity is below a certain tolerance. Friedrich et al.^23^ employed the root-mean-square norm of the scaled deviations of state variables between successive cycles below a tolerance limit as the CSS condition. Effendy et al.^32^ recently proposed a rigorous approach for defining CSS by reducing the differences between the current and CSS states of axial profiles below a set tolerance limit without requiring prior knowledge of the CSS itself.

This study considers that the CSS is attained when two conditions are met: (1) the maximum squared sum of the difference in state variables between two successive cycles, evaluated over all cycle steps and multiplied by 100, is less than 10^–5^ (hereafter referred to as the normalized residual error, see eq 2), and (2) the overall mass balance error remains below 0.5%. Note that the CSS criteria is based on strict tolerances which ensures a stable process performance.^7^

$$\mathrm{Normalized}\;\mathrm{residual}\;\mathrm{error}\;(-)=\max_{j}(100\sum_{i}^{M}(\Delta_{i,n}^{\left(j\right)}-\Delta_{i,n-1}^{\left(j\right)}{)}^{2})$$ 2

where Δ_*n*_^(*j*)^ and Δ_*n*–1_^(*j*)^ are the vectors of normalized state variables for step *j* at cycle *n* and *n* – 1, and *i* is an index that represents that runs over the *M* components of the ***S***.

## 3 Vector Acceleration Methods

This section presents acceleration methods for enhancing the convergence of fixed-point iterative vector sequences arising from successive substitution. Specifically, these methods rely on transformation (or extrapolation) of the original sequence into a new one that converges more rapidly under suitable conditions. Various well-established extrapolation techniques exist for solving nonlinear equations with one variable. These include Aiken’s Δ^2^ process,^21^ Steffensen’s method^27^ (a recursive formulation of Aiken’s Δ^2^), Anderson acceleration,^36^ and Wynn-ϵ algorithm^24^ (a recursive implementation of the Shanks transformation^37^). The discussion that follows provides a brief introduction to these acceleration schemes, starting from basic scalar sequences and their extensions to vector sequences. While some of these methods are widely popular in the broader numerical analysis and applied mathematics literature, their basic definitions and extensions to vector formulations are presented here to establish the necessary theoretical background relevant to the present study. Interested readers are referred to the works of Brezinski^25,38^ and more recent contributions by Ramière and Helfer,^28^ as well as Deng,^29^ for a detailed overview of these methods and their complete formulations.

### 3.1 Scalar Sequences

As a starting point, consider a scalar nonlinear fixed-point problem, *x*_*n*+1_ = *f*(*x*_*n*_), x∈R , and f:R→R , which can be reformulated as a root-finding problem under the assumption that *x*_*n*+1_ = *f*(*x*_*n*_) converges to a fixed point:

g(x)=f(x)−x 3

such that the fixed point x* = *f*(x*) is also a root of *g*(x*) = 0.

A common approach for solving eq 3 is Newton’s method,

$$x_{n+1}=x_{n}-\frac{g\left(x_{n}\right)}{g^{\prime }\left(x_{n}\right)}$$ 4

which provides a quadratic and faster convergence to solutions compared to FPIs. As discussed in the Introduction, the main drawback of Newton’s method as a convergence accelerator is the prior knowledge of first derivatives or Jacobian, i.e., *g′*(*x*_*n*_), which is not straightforward to calculate for many systems at relevant scale.

Alternatively, Newton’s method can be replaced with “derivative-free” sequence acceleration methods, such as Aitken’s Δ^2^ method or its recursive implementation of Steffensen’s method. Aiken’s Δ^2^ or Steffensen’s formulation for a scalar nonlinear fixed-point equation, *x* = *f*(*x*) can be represented as:

$$x_{n+1}=x_{n}-\frac{{\left(f\left(x_{n}\right)-x_{n}\right)}^{2}}{f\left(f\left(x_{n}\right)\right)-2f\left(x_{n}\right)+x_{n}}=x_{n}-\frac{u^{2}}{w}$$ 5

where the scalar variables *u*, *v*, and *w* are defined as *u* = *f*(*x*_*n*_) – *x*_*n*_, *v* = *f*(*f*(*x*_*n*_)) – *f*(*x*_*n*_), and *w* = *v* – *u*, respectively. The equivalent versions of eq 5 can also be expressed as,

$$x_{n+1}=f\left(x_{n}\right)-\frac{uv}{w}$$ 6

$$x_{n+1}=f\left(f\left(x_{n}\right)\right)-\frac{v^{2}}{w}$$ 7

Note that Steffensen’s method can be obtained by approximating the gradient *g′*(*x*_*n*_) in Newton’s method as

$$g^{\prime }\left(x_{n}\right)=\frac{g\left(x_{n}+g\left(x_{n}\right)\right)-g\left(x_{n}\right)}{g\left(x_{n}\right)}$$ 8

Notably, the application of Steffensen’s acceleration requires two new function evaluations (or successive substitution iterations) prior to its implementation.

### 3.2 Vectorization

Practical problems, such as adsorption process simulations, often deal with many unknown variables and systems of nonlinear PDEs. The spatial discretization of PDEs introduces a vector of state variables (Δ) across the column. Recall eq 1 that represents the vector fixed-point sequence. To accelerate the convergence of such vector sequences, several extrapolation schemes have been developed,^24,28,29,39–41^ many of which are extensions of scalar formulations. Among these, Steffensen’s method can be generalized to many variants of vectorization and will be the basis of this study.

To extend Steffensen’s scalar formulation into a vector form for a fixed-point sequence $\Delta_{n+1}=F\left(\Delta_{n}\right)$ , the scalar variables (*u*, *v*, and *w*) are first replaced by the vector counterparts ( $u=F\left(\Delta_{n}\right)-\Delta_{n}$ , $v=F\left(F\left(\Delta_{n}\right)\right)-F\left(\Delta_{n}\right)$ , **w** = **v** – **u**). Then, the further transformation involves the following considerations:^29^

1. When multiplying two vectors (**x**, **y**), the inner product of **x** ^*T*^ **y** (or **y** ^*T*^ **x**) is used to ensure scalar outputs.
2. The scalar denominator in eqs 5–7 is substituted with a suitable vector inverse, which can be expressed using either the Brezinski^42^ or Samelson^24,43^ inverse. For a pair of real vectors **x** and **y** such that **x** ^*T*^ **y** ≠ 0, the Brezinski inverse^42^ involves defining the inverse of a vector **x** via the inner product of another vector **y**:
$$x^{-1}=\frac{y}{y^{T}x}$$ 9
  The Samelson inverse^24,43^ is regarded as the special case of Brezinski inverse when **x** = **y,** and **x** ^–1^ definition involves the Euclidean norm of **x** as follows:
$$x^{-1}=\frac{x}{∥x{∥}^{2}}$$ 10

Building upon this, the scalar formulation in eq 5 can be transformed to a vector form using the Brezinski inverse, defined as **w** ^–1^ = **u**/**u** ^*T*^ **w**:

$$\begin{array}{rll} \Delta_{n+1} & =\Delta_{n}-∥u{∥}^{2}w^{-1} & \\ & =\Delta_{n}-\frac{∥u{∥}^{2}}{u^{T}w}u \end{array}$$ 11

where **u** ^*T*^ **w** is the inner product between the two vector variables. The ratio ∥**u**∥^2^/**u** ^*T*^ **w** is a scalar parameter, which can be denoted as λ_*n*_.

For the sake of brevity, only one example of transforming the scalar formulation into its vector form has been provided above. However, by combining the scalar expressions in eqs 5–7 with two alternative definitions of vector inverse, multiple variants of the vector variable Steffensen’s acceleration methods have been proposed.^29^ These vectorized formulations are summarized in Table 1. For further examples on the derivations of other variants, see Deng^29^ and Ramiere and Helfer.^28^ Similar to the work of Deng,^29^ the acceleration variants are classified into different classes for improved understanding.

**Table 1:** Different Variants of Vector Extensions of Steffensen’s Method Proposed in the Literature^28,29^ ^a^

**method**	**new iterate**	**scalar parameter**	**reference**
Class U methods			
Lemaréchal	Δ_*n*+1_ = Δ_*n*_ – λ_*n*_ **u**	$\lambda_{n}=\frac{u^{T}w}{∥w{∥}^{2}}$	^44^
Sedogbo	Δ_*n*+1_ = Δ_*n*_ – λ_*n*_ **u**	$\lambda_{n}=\frac{u^{T}v}{v^{T}w}$	^45^
A1	Δ_*n*+1_ = Δ_*n*_ – λ_*n*_ **u**	$\lambda_{n}=\frac{∥u{∥}^{2}}{u^{T}w}$	^29^
A4	$\Delta_{n+1}=F\left(F\left(\Delta_{n}\right)\right)-\lambda_{n}u$	$\lambda_{n}=\frac{∥v{∥}^{2}}{u^{T}w}$	^29^
Class V methods			
Irons and Tuck	$\Delta_{n+1}=F\left(\Delta_{n}\right)-\lambda_{n}v$	$\lambda_{n}=\frac{u^{T}w}{∥w{∥}^{2}}$	^39^
B1	Δ_*n*+1_ = Δ_*n*_ – λ_*n*_ **v**	$\lambda_{n}=\frac{∥u{∥}^{2}}{v^{T}w}$	^29^
Zienkiewicz-Lohner	$\Delta_{n+1}=F\left(\Delta_{n}\right)-\lambda_{n}v$	$\lambda_{n}=\frac{u^{T}v}{v^{T}w}$	^46^
Graves-Morris	$\Delta_{n+1}=F\left(\Delta_{n}\right)-\lambda_{n}v$	$\lambda_{n}=\frac{u^{T}v}{u^{T}w}$	^40,47^
Class W methods			
C1	Δ_*n*+1_ = Δ_*n*_ – λ_*n*_ **w**	$\lambda_{n}=\frac{∥u{∥}^{2}}{∥w{∥}^{2}}$	^29^
C2	$\Delta_{n+1}=F\left(\Delta_{n}\right)-\lambda_{n}w$	$\lambda_{n}=\frac{u^{T}v}{∥w{∥}^{2}}$	^29^
Macleod M6	$\Delta_{n+1}=F\left(F\left(\Delta_{n}\right)\right)-\lambda_{n}w$	$\lambda_{n}=\frac{∥v{∥}^{2}}{∥w{∥}^{2}}$	^41^
vector ϵ_2_	$\Delta_{n+1}=F\left(\Delta_{n}\right)-\lambda_{n}w+\eta_{n}u$	$\lambda_{n}=\frac{∥u{∥}^{2}}{∥w{∥}^{2}}$	^24,41^
		$\eta_{n}=\frac{∥v{∥}^{2}}{∥w{∥}^{2}}$	

a These variants are investigated for accelerating adsorption process simulations in the present study.

Unlike the scalar case, where the derivative term can be eliminated through second-order differences, there is no unique way to extend Steffensen’s method to vector sequences. In multiple dimensions, the vector differences **u**, **v**, and **w** obtained from a small number of function evaluations do not provide sufficient information for reconstructing the full Jacobian matrix. All vector extensions of the Steffensen’s method considered therefore define a scalar parameter λ_*n*_ (see Table 1) to approximate the effect of the inverse Jacobian along one search direction at a time, typically associated with the dominant eigenmode of the Jacobian. Different variants in Table 1 compute λ_*n*_ using different inner products of **u**, **v**, and **w** to project the same Jacobian information. In practice, these are not always equivalent, as each definition of λ_*n*_ represents a particular choice of search direction, the inner products used to define, and the sensitivity of those inner products to conditioning and noise. As a result, the convergence behavior and the numerical stability differs from variant to variant. Given that errors decay at different rates along different eigen directions, no single λ_*n*_ can accelerate all error components simultaneously; some may even slow down or become unstable. Consequently, variants whose search direction aligns better with the dominant error components typically converge faster. Because the dynamics of different adsorption processes to CSS vary with the regeneration method or initial guess and it is not known a priori which vector variant best suits each case, all variants are systematically examined in the present study.

To implement vector acceleration methods in adsorption process simulations, at least two FPIs are necessary based on eq 5, or its variants in eqs 6 and 7. Initially, the process simulations are run using successive substitution for the first three cycle iterations, and during the fourth cycle iteration, any of the vector acceleration methods from Table 1 is invoked. After this iteration, the acceleration is invoked after every three FPIs in between. Moreover, if the residual error converges to 1 × 10^–4^ during the process simulations, then the acceleration methods are no longer invoked and the cycles are repeated until CSS is used using successive substitution alone. This is to ensure that the residual error does not diverge and oscillate near the CSS solution. An empirical study is also carried out in Section 5.3 to understand the impact of the number of FPIs between two vector acceleration implementations. The computational times are recorded on a Dell Precision tower computer, and for the sake of clarity, the computational times are reported as a percentage of the reference process simulation using successive substitution.

### 3.3 Performance Metrics for Acceleration Methods

To enable a systematic comparison of different vector acceleration methods, we considered three performance metrics that capture both convergence efficiency and behavior: (1) the number of iterations required to reach CSS, (2) computational time expressed as a percentage of the reference process simulation using successive substitution, and (3) the average logarithmic contraction ratio (Λ̅), which quantifies the overall average contractivity along the convergence trajectory and is defined as:

$$\begin{array}{cc} \overset{-}{\Lambda }=\frac{1}{N}\overset{N}{\underset{n=1}{\sum }}\log\left(\frac{E_{n+1}}{E_{n}}\right), & E_{n}=∥\Delta_{n+1}-\Delta_{n}{∥}_{\infty } \end{array}$$ 12

where Λ̅ represents the average exponential rate of contraction of the residual per iteration and can be interpreted as a finite-time Lyapunov exponent, analogous to those used to measure convergence or divergence in chaotic dynamical systems.^48^ Note that Λ̅ < 0 generally implies convergence. The more the negative Λ̅, the faster the average convergence, even if individual iterates along the trajectory path oscillate.

## 4 Test Cases

Three different adsorption processes, namely, VSA, TSA, and VTSA are considered to test the vector acceleration methods. These processes differ significantly in their cycle dynamics and convergence behavior toward CSS from an arbitrary initial condition. In VSA processes, the cycle dynamics are heavily influenced by the pressure swing between the adsorption and the desorption steps. Similarly, dynamics in TSA processes are driven by the temperature swing. VTSA combines both pressure and temperature swings, affecting its cycle dynamics. It is important to note, however, that apart from the adsorbent the influence of pressure or temperature alone does not always fully capture the cycle dynamics for nonlinear adsorption systems. Often, the interplay between pressure and temperature governs the convergence behavior, and the extent of their coupled effect varies, depending on the regeneration strategy.

In adsorption process simulations, the column is usually initialized as fully saturated with either the light product or the feed mixture and the cycle is iterated until the CSS is attained. From a heavy product recovery standpoint (all relevant test cases in this work), these two initializations represent opposite extremes, with actual CSS profiles generally falling between them. These standard initializations are widely considered because the best initial guess leading to minimum cycle iterations in CSS is usually unknown in advance, as it depends on operating conditions, step durations, and cycle configurations. Moreover, a “good” initial guess for one process simulation may perform poorly for another. To avoid such complexities, particularly in optimization routines, standard initial guesses are adopted in process simulations, even though they may sometimes lead to slower convergence. In this work, all simulations are performed with full saturation with the feed composition as the initial condition. Additionally, the best-performing acceleration methods are further tested by performing simulations initialized with the light product, and their convergence behavior is provided in the Supporting Information. Note that the operating conditions defined below for each adsorption cycle constitute the reference case.

### 4.1 Four-Step VSA Cycle

As a first case study, a four-step VSA cycle separating the binary feed mixture of CO_2_/N_2_ for postcombustion CO_2_ capture^9^ is considered. The feed composition consists of 15% CO_2_ and 85% N_2_ at ambient pressure, representative of dry flue gas from coal-fired or waste-to-energy power plants. Figure 2a illustrates a schematic of the four-step VSA cycle. The cycle consists of feed pressurization (FP), adsorption (ADS), cocurrent blowdown (BLO), and counter-current evacuation (EVAC) steps. We consider here commercial zeolite 13X as the adsorbent, which exhibits a strong and nonlinear CO_2_ isotherm; while 13X might not be the most performing adsorbent for a real application, its nonlinear behavior makes it a particularly good test case for evaluating vector acceleration methods in process simulations. The equilibrium behavior of CO_2_ and N_2_ on zeolite 13X is expressed using the competitive dual-site Langmuir isotherm, consistent with the reference work. Simulation parameters and isotherm data are obtained from Haghpanah et al.,^9^ and one of their operating conditions is used as a baseline for the process simulation. Specifically, the cycle operates at 1 bar during adsorption (*P*_H_), with a blowdown step vacuum (*P*_I_) of 0.2 bar and an evacuation vacuum (*P*_L_) of 0.1 bar. The interstitial feed velocity during adsorption is set to 1 m s^–1^. Step durations are 15 s for feed pressurization, 15 s for adsorption, 30 s for blowdown, and 40 s for evacuation.

**Figure 2:**
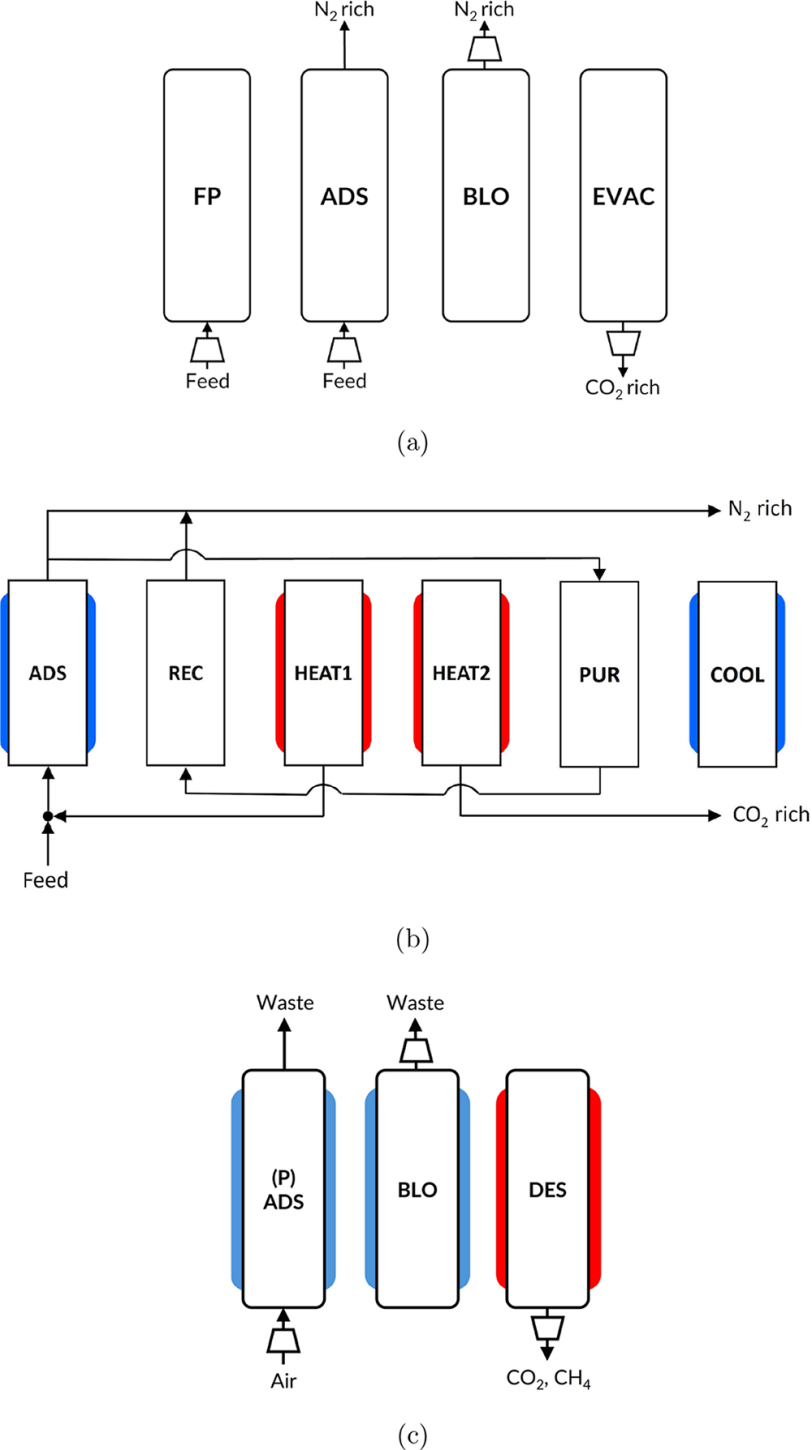
Different adsorption processes considered for testing vector acceleration methods. (a) Four-step vacuum swing adsorption cycle (adapted from Haghpanah et al.^9^ Copyright 2013 American Chemical Society), (b) six-step temperature swing adsorption cycle^7^ designed for postcombustion CO_2_ capture (adapted with permission from Joss et al.^7^ Copyright 2017 Elsevier), and (c) three-step vacuum temperature swing adsorption cycle designed for CO_2_ and CH_4_ enrichment (reproduced from Subraveti et al.^49^ under CC BY-NC-ND 4.0 license. Copyright 2025 Subraveti et al.).

### 4.2 Six-Step TSA Cycle

The second case study involves a six-step TSA cycle proposed by Joss et al.^7^ to separate CO_2_ from a binary feed mixture of 12% CO_2_ and 88% N_2_ using commercial zeolite 13X. Figure 2b shows the six-step TSA cycle, which consists of adsorption (ADS), recovery (REC), two heating (HEAT), purge (PUR), and cooling (COOL) steps. For simulating this process, the cycle operating conditions and the simulation parameters have been retrieved from Joss et al.^7^ The adsorption step occurs under a constant pressure of 1.3 bar and at an ambient temperature of 300 K. In the heating steps, the column is externally heated to 420 K to desorb CO_2_. The cycle is simulated for the following operating conditions: adsorption temperature *T*_COOL_ = 300 K, desorption temperature *T*_HEAT_ = 420 K, interstitial velocity *v*_0_ = 1.4 m s^–1^, step durations are 150 s for adsorption, 25 s for recovery and purge, 150 and 600 s for the first and second heating, and 600 s for cooling. It is important to note that TSA cycles generally have longer durations than VSA cycles, primarily due to the slower heat transfer dynamics.

### 4.3 Three-Step VTSA Cycle

The third case is a three-step VTSA cycle, as shown in Figure 2c, designed for enriching CH_4_ and CO_2_ separation from dilute air feeds.^49^ Specifically, the cycle aims to preconcentrate CH_4_ and CO_2_ from a feed mixture consisting of 200 ppm of CH_4_, 400 ppm of CO_2_, and the rest of the N_2_ at 1 bar and near-ambient temperatures. This case highlights the application of adsorption processes for the separation and purification of trace gas components, similar to the case of direct air capture. The cycle design was based on a hypothetical, exemplary adsorbent proposed by Sabatino et al.,^50^ for which the CO_2_ isotherm data were readily available. The CO_2_ isotherm is highly nonlinear, especially at low concentrations, to achieve the desired adsorption capacities. Since this is a novel separation at the nascent stage, Subraveti et al.^49^ carried out integrated adsorbent-process optimizations to determine the ideal CH_4_ and N_2_ isotherm properties for the same exemplary adsorbent; here, we use the same CH_4_ and N_2_ isotherms. All isotherms are represented using an extended Langmuir-Freundlich isotherm model, which effectively captures nonlinearity at low concentrations and accounts for the competition among multiple gas components. The necessary simulation and isotherm data for this case can be obtained from Subraveti et al.^49^ Finally, the following cycle operating conditions, which are reported by Subraveti et al.,^49^ are used in the simulation to test the acceleration methods: adsorption temperature *T*_COOL_ = 277.3 K, desorption temperature *T*_HEAT_ = 382.7 K, a blowdown vacuum pressure *P*_L_ = 0.03 bar, and an interstitial velocity *v*_0_ = 0.1 m s^–1^. Step durations are 16,188 s for adsorption and 9800 s for heating/desorption.

Moreover, we test the portability of the vector acceleration methods by implementing them in two different adsorption process simulation frameworks: one in Python 3.13 as reported in Subraveti et al.^49^ and one in Fortran 2023 as described, among many other sources, in Joss et al.^7^ This portability test is carried out exclusively for VTSA application. In the Fortran 2023 implementation, the process was simulated using an existing metal–organic framework, ATC-Cu,^51,52^ as the adsorbent, rather than the ideal hypothetical adsorbent employed in the Python implementation. The bed specifications are consistent with those reported in Sabatino et al.^50^

## 5 Results and Discussion

### 5.1 Comparative Performance of Acceleration Methods for the Reference Case

#### 5.1.1 Four-Step VSA Cycle

A comprehensive set of process simulations was conducted based on the reference operating conditions provided in Section 4.1 to evaluate all variants of vector acceleration methods listed in Table 1. To maintain readability in the plots, only the best-performing acceleration methods from each class are presented, and the comparative performances of the remaining methods can be found in Table 2. Figure 3a,b illustrates their convergence behavior from initial feed conditions to CSS by showing the maximum change in normalized state variables between successive iterations across different steps of the cycle (i.e., the normalized residual error), along with the mass balance error, respectively. The corresponding simulation times and process performance in terms of the CO_2_ purity and recovery obtained at CSS are provided in Table 2. Without any acceleration, the FPI using successive substitution required 340 cycles to reach the steady state (CSS), at which point both the mass balance closure and the normalized residual error were within 0.5% and 1× 10^–5^, respectively. To understand which state variables most strongly influence the convergence of this VSA cycle to CSS, we examined the variable-wise normalized residual norms across iterations (see Section S2.1 and Figure S1 in the Supporting Information). Among them, the solid-phase CO_2_ loading (*q*_CO_2__) was found to be the dominant, slowly converging variable, contributing on average 69% to the residuals over the cycle iterations. This is mainly due to the nonlinearity of the competitive dual-site Langmuir CO_2_ isotherm (eq S8 in Table S2), as expressed through the associated LDF equation (eq S5 in Table S1). The next most influential variable is the column temperature, accounting for an average residual contribution of 21%. As expected, distinct convergence behaviors are observed among the various Steffensen-type variants, as each method employs a different approximation of the Jacobian inverse through its choice of search direction and λ_*n*_. Among the acceleration methods evaluated, the fastest to converge were the Class V extrapolators: Zienkiewicz-Lohner and Graves-Morris, which reached CSS in 124 and 121 cycles, respectively. These methods offered almost 3× convergence speeds (121 vs 340) and more than 4× reduction in computational times compared with successive substitution. According to the third metric, average logarithmic contraction ratio, Zienkiewicz-Lohner and Graves-Morris yielded comparable values of Λ̅ = −0.045 and −0.046, respectively, both exceeding (in magnitude) those obtained with successive substitution and other acceleration variants. The superior performance of Zienkiewicz-Lohner and Graves-Morris methods for VSA simulations likely stems from the combination of the following three reasons: 1) accelerating the updated iterate $F\left(\Delta_{n}\right)$ , which is naturally closer to the solution than Δ_*n*_, 2) using $v=F\left(F\left(\Delta_{n}\right)\right)-F\left(\Delta_{n}\right)$ as the search direction that captures the change in F from the successive function evaluations may better align with the dominant eigenmode for this case, and 3) employing better-conditioned and well-scaled λ_*n*_. The variable-wise residual contributions obtained with Graves-Morris align very closely with those observed for the successive substitution, with *q*_CO_2__ remaining the slowest-converging variable. Additionally, their implementation was relatively straightforward. B1 method showed comparable but slightly slower convergence, likely due to the acceleration applied to the base iterate Δ_*n*_. In contrast, the Irons and Tuck method converged slowly due to poor conditioning of λ_*n*_, where small values of ∥**w**∥^2^ in the denominator possibly amplified numerical noise. This is reiterated with a lower negative value of Λ̅, indicating lower average contraction per iteration.

**Table 2:** Comparative Performance of Vector Acceleration Methods and Successive Substitution at CSS for the Four-Step VSA Cycle Based on the Reference Operating Condition Provided in Section 4.1 ^a^

	method	#cycles to CSS	comput. time (%)	Λ̅	CO_2_ purity (%)	CO_2_ recovery (%)
	successive	340	100	–0.018	79.7	37.3
	substitution					
U‐class	Lemaréchal	272	50	–0.023	79.7	37.3
	Sedogbo	164	57	–0.039	79.7	37.2
	A1	154	28	–0.031	79.7	37.3
	A4	155	29	–0.038	79.7	37.3
V‐class	Irons and Tuck	232	43	–0.027	79.7	37.3
	B1	126	45	–0.043	79.7	37.3
	Zienkiewicz-Lohner	124	24	–0.045	79.7	37.2
	Graves-Morris	121	23	–0.046	79.7	37.3
W‐class	C1					
	C2	500	164	–0.011	79.7	37.3
	M6	330	87	–0.019	79.7	37.3
	vector ϵ_2_	306	93	–0.020	79.7	37.3

a Note that Λ̅ is the average logarithmic contraction ratio.

**Figure 3:**
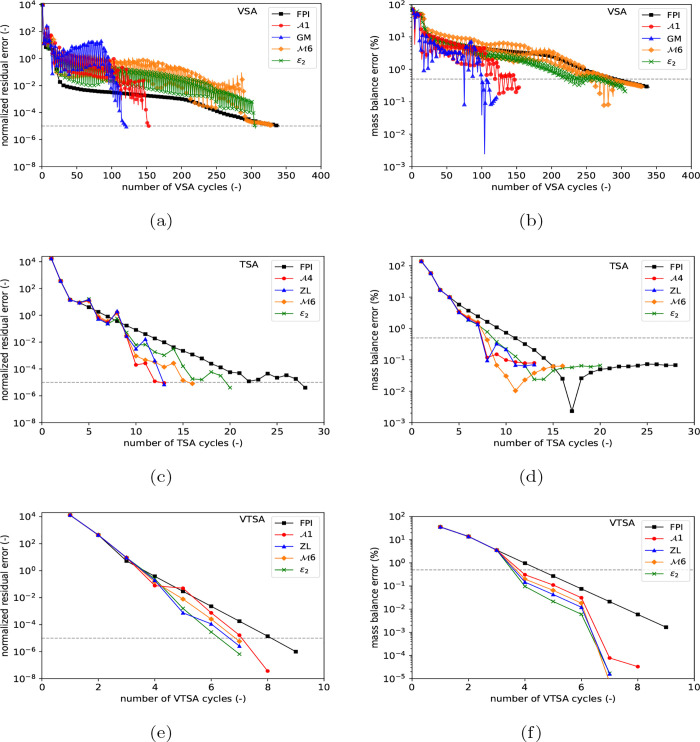
Convergence of normalized residual (left) and mass balance (right) errors from a feed mixture initial condition to CSS using best-performing vector acceleration methods and successive substitution for different adsorption cycles. (a) and (b) represent the four-step VSA cycle, (c) and (d) six-step TSA cycle, and (e) and (f) three-step VTSA cycle. The simulations are based on the operating conditions reported in Section 4. FPI: fixed-point iteration (or successive substitution), ZL: Zienkiewicz-Lohner, and GM: Graves-Morris.

On the other hand, Class U variants outperformed successive substitution as well, converging in 154–272 cycles (see Table 2). Particularly, A1 and A4 methods were competitive with Zienkiewicz-Lohner and Graves-Morris extrapolators, but Lemaréchal and Sedogbo variants were found to be slower than the Class V methods. For the latter two, the combination of weak-conditioned λ_*n*_ and limited directional information from one functional evaluation in the search direction, $u=F\left(\Delta_{n}\right)-\Delta_{n}$ , possibly resulted in slower error reductions. The poorest convergence was observed in class W and vector ϵ_2_, which required more number of cycle iterations to CSS. Notably, the C2 method did not converge within the maximum number of cycles set in the simulations. In fact, the average per-iteration contraction, as inferred from Λ̅, is lower than that obtained with successive substitution. Moreover, M6 took about 87% of the time of successive substitution to converge in 330 cycles. While class W methods leverage multipoint information in the **w** search direction, their poor convergence for this case may be attributed to numerically ill-conditioned λ_*n*_ with small ∥**w**∥^2^ in the denominator, amplifying the errors. The popular ϵ_2_ method also exhibited slow convergence because of poorly conditioned scalar values, requiring 306 cycles, and offered minimal computational speed-up compared with successive substitution.

In the convergence profiles shown in Figure 3a,b, invoking vector acceleration methods introduced oscillations during convergence. In some cases, such as the Class V variants, these oscillations facilitated faster convergence due to well-conditioned λ_*n*_, without causing significant divergence. Conversely, some other variants occasionally produced unstable extrapolations or physically infeasible guesses due to the ill-conditioned λ_*n*_. This introduced stiffness or instability into the ODE system, slowing down the integration and, in some instances, preventing convergence altogether (e.g., M6 method). As a result, although some methods required fewer cycles for CSS than successive substitution, the overall simulation times remained the same. Furthermore, it is important to note that acceleration methods converging in similar cycle iterations could still differ in the overall simulation time due to the differences in extrapolated guesses in each method and their impact on the ODE system stiffness and numerical stability.

Finally, A1 and Graves-Morris extrapolators are evaluated under light product initialization, where the simulation begins with the column fully saturated with light product until reaching CSS. The results confirm the merit of Graves-Morris method, enabling once again a nearly 3-fold faster convergence while reducing computational times by more than half. The detailed analyses are provided in Section S2.2 of the Supporting Information.

#### 5.1.2 Six-Step TSA Cycle

A similar experimental campaign was repeated for the six-step TSA cycle based on the reference operating conditions, and the convergence profiles from the best-performing acceleration methods from each class are shown in Figure 3c, with the corresponding mass balance errors in Figure 3d. The comparative performances of all vector acceleration methods for this case study are reported in Table 3. As can be seen from Figure 3c, the FPI needed 28 cycles to reach CSS, where the overall mass balance closure and the normalized residual error were within the set tolerances. An analysis of the variable-wise residual contributions (Figure S1) indicated that *q*_CO_2__ overwhelmingly dominates the residuals, accounting for an average of 89%. This pronounced effect can again be attributed to the nonlinearity of the competitive Sips CO_2_ isotherm (eq S10) reflected through the corresponding LDF equation (eq S5). Although TSA cycles generally converge to CSS in fewer iterations than PSA/VSA cycles, each TSA cycle iteration requires significantly more computational time. This is consistent with the physics and dynamics behind pressure- vs temperature swings. Among all the acceleration methods tested, A4 variant from Class U and Zienkiewicz-Lohner extrapolator from Class V demonstrated the fastest convergence, reaching CSS in 13 cycles instead of 28 (successive substitution) and taking only a third of the computational time. Based on the third metric, A4 variant clearly outperforms Zienkiewicz-Lohner method by achieving the highest negative value of Λ̅ = −0.68, indicating the fastest average convergence per iteration. Class U methods, A1 and Sedogbo extrapolator, also converged in fewer cycles (16–18) compared to successive substitution, and improved the simulation times by ∼50%. Other Class V methods generally took 17–19 cycles to converge to CSS, outperforming successive substitution in terms of cycle iterations and reduced the computational times by almost half. ϵ_2_ performed well in terms of reducing computational times by doubling the computational savings and reaching CSS in 20 cycles. Among Class W methods, C2 and M6 offered 2× computational gains and converged in 19 and 16 cycles, respectively. Contrarily, the C1 method was an outlier, taking 29 cycles, which is more than successive substitution, to reach CSS. Unlike the VSA case, many of Class U , V , and W variants showed comparable convergence, which could be due to relatively weaker nonlinearity in the fixed-point mapping F(Δ)−Δ . This means that search directions reasonably align well with the dominant eigenmode, moreover, **v** and **w** adding little extra directional information. Nonetheless, their relative performance still depends on how well λ_*n*_ is scaled.

**Table 3:** Comparative Performance of Vector Acceleration Methods and Successive Substitution at CSS for the Six-Step TSA Cycle Based on the Reference Operating Condition Provided in Section 4.2 ^a^

	method	#cycles to CSS	comput. time (%)	Λ̅	CO_2_ purity (%)	CO_2_ recovery (%)
	successive	28	100	–0.22	93.1	99.3
	substitution					
U‐class	Lemaréchal	22	66	–0.39	93.1	99.3
	Sedogbo	18	54	–0.37	93.1	99.3
	A1	16	45	–0.47	93.1	99.3
	A4	13	35	–0.68	93.1	99.3
V‐class	Irons and Tuck	18	57	–0.36	93.1	99.3
	B1	19	52	–0.41	93.1	99.3
	Zienkiewicz-Lohner	13	31	–0.38	93.1	99.3
	Graves-Morris	17	49	–0.37	93.1	99.3
W‐class	C1	29	111	–0.24	93.1	99.3
	C2	19	50	–0.29	93.1	99.3
	M6	16	50	–0.43	93.1	99.3
	vector ϵ_2_	20	54	–0.30	93.1	99.3

a Note that Λ̅ is the average logarithmic contraction ratio.

Remarkably, when re-evaluated under light product initialization, A4 and Zienkiewicz-Lohner extrapolators confirm the improved performance: the computational times is reduced by half with faster convergence speeds (see Section S2.2 of the Supporting Information).

When the accelerators are incorporated into the TSA process simulations, fewer oscillations with lower amplitudes are observed compared with VSA simulations. This difference likely stems from the slower heat transfer dominating the TSA cycle dynamics as the system progresses from an arbitrary initial state to CSS. The slow heat transfer causes small axial profile changes between the cycle iterations and, thus, limits oscillations during extrapolations. In contrast, VSA cycle dynamics is heavily influenced by faster mass transfer, resulting in more pronounced profile changes after each iteration. This leads to higher oscillations when extrapolation techniques are applied. Finally, it is worth reiterating that the implementation of the investigated acceleration methods is straightforward.

#### 5.1.3 Three-Step VTSA Cycle

Figure 3e,f illustrates the CSS convergence of the normalized residual and the mass balance error of the best-performing vector acceleration methods from each category for the three-step VTSA cycle simulations. Table 4 compares the performances of all variants with successive substitutions. The simulations were based on the operating conditions described in Section 4.3. Unlike the other two case studies that involve processes enabling high purities and recoveries, this case study presents a separation problem without a recovery constraint. By relaxing the high recovery requirement, the target gas components adsorb throughout the entire column to enable the desired purities from the dilute concentration feeds. This makes the feed initialization a suitable starting point for the process simulations. As cycles repeat during the simulation, state variables adjust toward the CSS condition in fewer iterations than the conventional separations. The process simulation required 9 cycle iterations using successive substitution to reach CSS. In contrast to VSA and TSA cases, the variable-wise residual contributions for the VTSA simulation (Figure S1) revealed that the gas-phase CO_2_ and CH_4_ compositions (eq S1) each account for ∼50% of the total residuals. Although the CO_2_ and CH_4_ isotherms are fairly nonlinear at the dilute feed concentrations considered, the mass transfer coefficients are orders of magnitude lower than those in VSA and TSA simulations. As a result, mass transfer resistances dampen the effect of isotherm nonlinearity, leading to faster convergence of the solid-phase loadings relative to other state variables. The process simulations employing the acceleration methods took 7–8 cycle iterations. While the improvement in cycle iterations is marginal since the acceleration method was applied only twice before reaching CSS, acceleration methods, particularly methods Zienkiewicz-Lohner, M6 , and ϵ_2_, offered almost 30% lower computational times than successive substitution in converging to CSS. This notable increase in computational efficiency, despite only a slight reduction of 1–2 cycle iterations, can be attributed to the extrapolated solution estimates that may have facilitated faster ODE integration in subsequent cycles, compared to the solutions encountered in successive substitution. When evaluated using the third metric, Λ̅, ϵ_2_ exhibited the highest average contractivity per iteration. In summary, Zienkiewicz-Lohner resulted in a slightly lower computational time (72%) but somewhat weaker contraction (Λ̅ = −1.16), whereas ϵ_2_ obtained stronger contraction (Λ̅ = −1.30) with modestly more computational time (77%). It is worth reiterating that these observations are specific to this reference simulation and may not necessarily generalize across other operating conditions.

**Table 4:** Comparative Performance of Vector Acceleration Methods and Successive Substitution at CSS for the Three-Step VTSA Cycle Based on the Reference Operating Condition Provided in Section 4.3 ^a^

	method	#cycles to CSS	comput. time (%)	Λ̅	CH_4_ purity (%)	CH_4_ recovery (%)
	successive	9	100	–0.93	32.0	84.4
	substitution					
U‐class	Lemaréchal	8	158	–0.97	32.0	84.4
	**Sedogbo**	8	93	–0.97	32.0	84.4
	A1	8	87	–0.97	32.0	84.4
	A4	7	101	–1.10	32.0	84.4
V‐class	Irons and Tuck	7	134	–1.16	32.0	84.4
	B1	8	97	–0.90	32.0	84.4
	Zienkiewicz-Lohner	7	72	–1.16	32.0	84.4
	Graves-Morris	7	82	–1.16	32.0	84.4
W‐class	C1	8	87	–0.87	32.0	84.4
	C2	8	90	–0.94	32.0	84.4
	M6	7	78	–1.08	32.0	84.4
	vector ϵ_2_	7	77	–1.30	32.0	84.4

a Note that Λ̅ is the average logarithmic contraction ratio.

To verify the portability of the vector acceleration methods, we applied them to the same VTSA case by using a different adsorption process simulator. In this instance, the model was implemented in Fortran 2023 with a different time integrator (LSODE^31^), as described in Joss et al. and Sabatino et al.^7,50^ The performance of Graves-Morris and Irons and Tuck variants from Class V were compared with successive substitution. Results are shown in Figure 4. While the best-performing accelerators differ between implementations, both the reduction in computing time and the decrease in cycles required to reach CSS are consistently observed. In fact, a more significant speed-up is achieved in this case due to the larger number of iterations (25 cycles solved in 48 s) required by this process simulator when using fixed substitution. More specifically, Graves–Morris achieved CSS in 30 s using 14 cycles, while the Irons and Tuck reached CSS in 31 s using 15 cycles, corresponding to 63 and 64% of the computational time required by the successive substitution, respectively. These results reinforce that the vector acceleration methods are portable and constitute a powerful tool that can be readily incorporated into existing fixed-bed process simulators.

**Figure 4:**
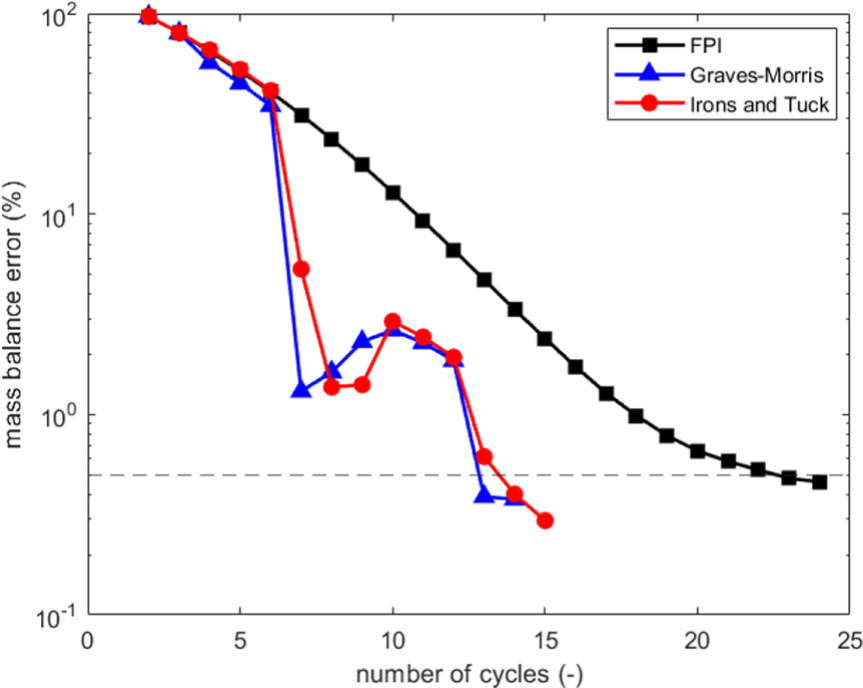
Convergence of the mass balance error from a feed mixture initial condition to CSS for a three-step VTSA cycle, comparing Class V acceleration methods (Graves-Morris and Irons and Tuck) with successive substitution. The VTSA cycle is simulated using an adsorption process model implemented in Fortran 2023, with LSODE as the solver.^7,31,50^

### 5.2 Comparative Performance of Acceleration Methods across Multiple Simulations

Previously, the acceleration methods were tested on process simulations using a fixed reference set of operating conditions. While it demonstrated their potential in speeding up adsorption process simulations, a broader assessment is required to quantify their computational advantages by evaluating them across the entire process design space. This is necessary because the convergence behavior toward the CSS depends not only on the initial guess used to start the simulation but also on the operating conditions (which influence the position of mass and heat transfer fronts within the column). To address this, we evaluate a large set of simulations for the four-step VSA cycle, which proved to be the most challenging to converge to CSS. Two vector acceleration variants, namely Graves-Morris and A1 , each identified as top-performing methods from different classes in Section 5.1.1, along with the popular ϵ_2_ algorithm, were selected for the analysis. A total of 500 unique operating conditions covering the whole design space were generated using Latin hypercube sampling. The design variables varied over the following ranges: *P*_I_: 0.03–0.7 bar, *P*_L_: 0.03–0.1 bar, *v*_0_: 0.01–0.1 m s^–1^, *t*_ADS_: 20–100 s, *t*_BLO_: 30–200 s, and *t*_EVAC_: 30–200 s.

Table 5 reports the average computational times of each method relative to successive substitution. All vector acceleration variants outperformed the successive substitution. Among them, the Graves-Morris extrapolator was consistently the fastest, achieving an average computational speed-up of 2× and in some cases reducing computational time by nearly 4-fold. A1 and the ϵ_2_ algorithm provided average computational gains of ∼60 and ∼30%, respectively. Overall, this analysis confirms that vector acceleration methods, particularly Graves-Morris, can improve process simulation times by at least a factor of 2 and in some cases by more than four times, highlighting their potential for accelerating VSA process design and, even more importantly, optimization.

**Table 5:** Average Computational Times Needed for Vector Acceleration Methods and Successive Substitution to Simulate Four-Step VSA Cycle across 500 Unique Operating Conditions

method	computational time (%)
successive substitution	100
A1	63
Graves-Morris	51
vector ϵ_2_	77

Building on the earlier analysis, which assessed acceleration methods across a range of operating conditions to provide a broader view of the associated computational gains, a local sensitivity analysis is performed to further quantify the improvement in convergence achieved with vector acceleration methods relative to successive substitution by examining the impact of individual operating variables, which also influence the convergence to CSS. For this analysis, the four-step VSA cycle was considered as the test case, alongside the Graves-Morris variant as the vector acceleration method. Each operating variable was perturbed ±20% of its baseline value in Section 4.1 while keeping all other variables constant.

In this sensitivity analysis, the impact of individual operating variables on CSS convergence, along with the relative performance of the Graves–Morris accelerator and successive substitution, is evaluated by using the three performance metrics. While the full results are provided in Section S2.3 of the Supporting Information, the Graves–Morris method consistently achieves more than 2.5× faster convergence and up to 3 times reduction in computational time, along with approximately 40% more contraction per iteration, compared to successive substitution when each operating variable is individually perturbed by ±20% relative to its nominal value. In terms of parameter sensitivity, vacuum pressure during the evacuation and blowdown steps exerts the strongest influence on CSS convergence, followed by the interstitial feed velocity.

### 5.3 Impact of the Number of FPIs

As noted earlier, at least two FPIs are theoretically required for the vector acceleration methods discussed here. In practice, however, the number of FPIs between successive accelerations can be varied to balance the convergence speed and computational cost. Fewer than two FPIs may lead to numerical instability or longer cycle iterations due to the overuse of acceleration methods, while more FPIs reduce the frequency of acceleration, potentially slowing convergence. This section systematically examines the effect of varying the number of FPIs between successive accelerations from 2 to 5 on the overall convergence to CSS in VSA and TSA cycle simulations. For the four-step VSA cycle case, A1 and Graves-Morris extrapolators, which showed superior performance in their categories, are selected to understand their distinct convergence behaviors. Similarly, A4 and Zienkiewicz-Lohner methods are investigated for the TSA cycle. Note that the simulations conducted here are based on the reference operating conditions reported in Section 4.

Figure 5 shows the convergence of the normalized residual error for different numbers of FPIs between the acceleration steps, with corresponding computational times in Table S6 in the Supporting Information. In VSA simulations, A1 variant, with 2 FPIs in between, converges slowly, as seen in Figure 5a, requiring 340 cycle iterations and 82% of the time of successive substitution. Increasing the number of iterations from 2 to 3 reduces the number of cycles to 154, cutting computational times by more than a third. For 4 and 5 iterations, cycles increase slightly to 157 and 165, but computational time also increases to 49 and 56% of successive substitution, respectively. For the Graves-Morris method in Figure 5b, convergence is fastest with three FPIs (121 cycles), offering slightly more than 4× computational savings, while further increasing the iterations led to slow convergence and moderately longer times. For TSA simulations using A4 and Zienkiewicz-Lohner methods, applying acceleration after every three FPIs achieves the fastest convergence with significant computational savings, as shown in Figure 5c,d, respectively.

**Figure 5:**
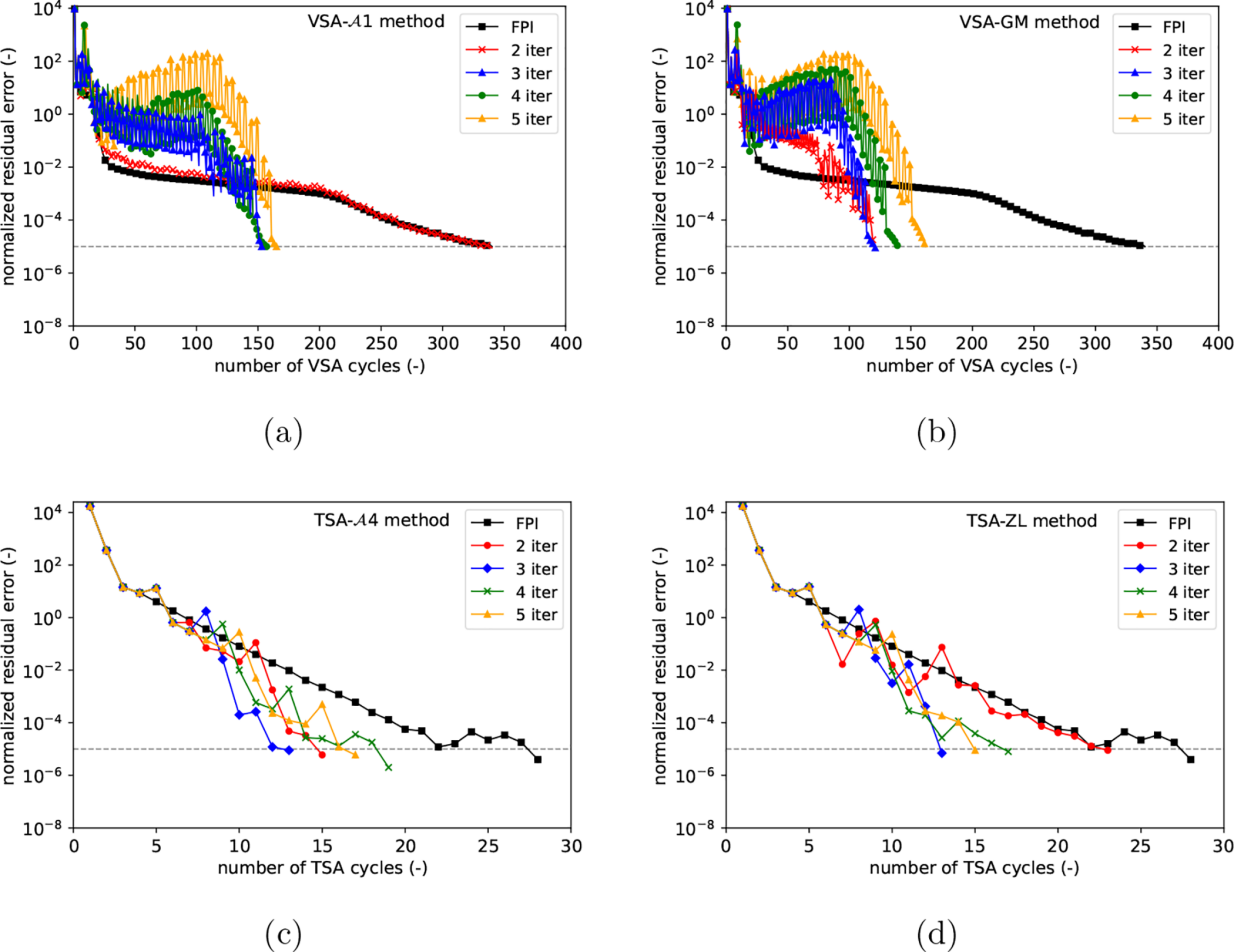
Effect of the number of FPIs in between consecutive accelerations on the convergence of normalized residual (left) and mass balance (right) errors for (a) and (b) four-step VSA and (c) and (d) six-step TSA cycles, respectively. A1 and Graves-Morris (GM) extrapolators are considered for the VSA case, whereas A4 and Zienkiewicz-Lohner (ZL) methods are used in the TSA analysis.

## 6 Conclusions

Simulating cyclic adsorption processes is computationally demanding due to iterative solving of nonlinear PDEs for each cycle until CSS is reached. Although previous efforts have sought to accelerate CSS convergence, the problem of CSS determination remains a major limitation in adsorption process simulations. To address this challenge, this study thoroughly investigates the application of vector acceleration methods for accelerating CSS convergence and reducing computational costs. Derived originally from Steffensen’s method for accelerating root-finding problems, these extrapolators have the potential to offer quadratic convergence near the CSS solution, similar to Newton’s method, but without the need for prior knowledge of first derivatives or Jacobian.

Several variants of vector acceleration methods are systematically examined for their ability to accelerate CSS convergence by considering three different adsorption processes, namely, four-step VSA and six-step TSA cycles designed for postcombustion CO_2_ capture, and a three-step VTSA cycle designed for CH_4_ upgrading from dilute atmospheric sources. Three performance metrics, namely, iterations to CSS convergence, computational time, and average logarithmic contraction ratio, were considered for assessing the acceleration methods. The results demonstrated that these methods, particularly the Graves-Morris extrapolator, offered nearly 3-fold convergence speeds in the reference VSA cycle simulation by reaching the CSS solution in only a quarter of the computational time needed for successive substitution. When the assessment was extended to 500 unique simulations, which would normally span several hours to a few days, the methods consistently achieved an average computational savings of at least a factor of 2. Similar trends were observed in TSA cycle simulations, where certain variants of these methods doubled the convergence speeds and cut the computational time to one-third of that needed with successive substitution. In the VTSA case, the acceleration methods were invoked fewer number of times, owing to the nature of the separation, achieving marginal improvement in cycle iterations yet reducing the computational times by 35%. Notably, the vector acceleration methods demonstrated robustness with respect to the adsorption process simulation framework: the VTSA acceleration was applied to a Fortran-based model, showing remarkable acceleration in line with the results of the Python model.

Overall, vector acceleration methods can significantly cut computational costs, particularly in large-scale process optimizations that require probing thousands of operating conditions. Importantly, these methods are straightforward to implement within existing process simulators and have a limited number of tuning parameters, most notably the number of fixed point iterations between two consecutive accelerations.

Although vector acceleration methods improved the rate of CSS convergence, the associated reduction in computational costs was not significantly greater than that of other acceleration strategies suggested in the literature. As noted previously, these methods perform well in the quasi-linear region near the CSS solution, suggesting that combining vector acceleration methods with smarter initialization rather than relying on standard starting points could further improve convergence, warranting future investigation. Finally, integrating vector acceleration with machine-learning-based approaches may provide a promising path toward even faster CSS convergence.

## Supplementary Material



## Notes

Tables of model equations and competitive isotherm models; details on state variable influence on convergence, with a figure illustrating the fractional contribution of normalized residuals of each variable; figures comparing convergence behaviors of vector acceleration methods and successive substitution from light product initialization to CSS, along with tables reporting corresponding computational times and process performances; results of local sensitivity analysis, including a figure showing the influence of input variable perturbations on acceleration efficiency and the sensitivity of each variable on performance metrics; and a table showing the impact of the number of fixed-point iterations between consecutive accelerations on convergence speed and computational times for both four-step VSA and six-step TSA cycles (PDF)
