# Supplementary material for: Vector Acceleration Methods for Faster Convergence of Cyclic Steady State in Adsorption Process Simulations
Source: Ind Eng Chem Res. 2026 Apr 28;65(18):9656–69. doi: 10.1021/acs.iecr.6c00407 (PMC13186178; doi:10.1021/acs.iecr.6c00407)
Supplement: Supplementary file 1 [file ie6c00407_si_001.pdf]

# Supporting Information
# Vector acceleration methods for faster convergence of cyclic steady state in adsorption process simulations

Sai Gokul Subraveti[1,*], Kian Karimi[2], and Matteo Gazzani[2,3]

[1]*SINTEF Energy Research, Sem Sælands vei 11, Trondheim, 7034, Norway*
[2]*Utrecht University, Copernicus Institute of Sustainable Development, Princetonlaan 8a, 3584 CB Utrecht, the Netherlands*
[3]*Sustainable Process Engineering, Chemical Engineering and Chemistry, Eindhoven University of Technology, 5612 AP Eindhoven, the Netherlands*
[*]*Corresponding author. E-mail: sai.gokul.subraveti@sintef.no (Sai Gokul Subraveti)*

# S1 Modeling

Table S1: Model equations.

component mass balance

$$\frac{\partial y_{\mathrm{i}}}{\partial t}+\frac{y_{\mathrm{i}}}{P}\frac{\partial P}{\partial t}-\frac{y_{\mathrm{i}}}{T}\frac{\partial T}{\partial t}=\frac{T}{P}D_{\mathrm{L}}\frac{\partial}{\partial z}\left(\frac{P}{T}\frac{\partial y_{\mathrm{i}}}{\partial z}\right)-\frac{T}{P}\frac{\partial}{\partial z}\left(\frac{y_{\mathrm{i}}P}{T}v\right)-\frac{RT}{P}\frac{1-\varepsilon}{\varepsilon}\frac{\partial q_{\mathrm{i}}}{\partial t} \tag{S1}$$

total mass balance

$$\frac{1}{P}\frac{\partial P}{\partial t}-\frac{1}{T}\frac{\partial T}{\partial t}=-\frac{T}{P}\frac{\partial}{\partial z}\left(\frac{P}{T}v\right)-\frac{RT}{P}\frac{1-\varepsilon}{\varepsilon}\sum_{i=1}^{n_{\mathrm{comp}}}\frac{\partial q_{\mathrm{i}}}{\partial t} \tag{S2}$$

column energy balance

$$\begin{aligned}\left[\frac{1-\varepsilon}{\varepsilon}\left(\rho_{\mathrm{s}}C_{\mathrm{p,s}}+C_{\mathrm{p,a}}\sum_{i=1}^{n_{\mathrm{comp}}}q_{\mathrm{i}}\right)\right]\frac{\partial T}{\partial t}&=\frac{K_{\mathrm{z}}}{\varepsilon}\frac{\partial^2 T}{\partial z^2}-\frac{C_{\mathrm{p,g}}}{R}\frac{\partial P}{\partial t}-\frac{C_{\mathrm{p,g}}}{R}\frac{\partial}{\partial z}\big(vP\big)\\&-\frac{1-\varepsilon}{\varepsilon}C_{\mathrm{p,a}}T\sum_{i=1}^{n_{\mathrm{comp}}}\frac{\partial q_{\mathrm{i}}}{\partial t}+\frac{1-\varepsilon}{\varepsilon}\sum_{i=1}^{n_{\mathrm{comp}}}\left(\Big(-\Delta H\Big)\frac{\partial q_{\mathrm{i}}}{\partial t}\right)-\frac{2h_{\mathrm{in}}}{\varepsilon r_{\mathrm{in}}}\big(T-T_{\mathrm{w}}\big)\end{aligned} \tag{S3}$$

wall energy balance

$$\rho_{\mathrm{w}}C_{\mathrm{p,w}}\frac{\partial T_{\mathrm{w}}}{\partial t}=K_{\mathrm{w}}\frac{\partial^2 T_{\mathrm{w}}}{\partial z^2}+\frac{2r_{\mathrm{in}}h_{\mathrm{in}}}{r_{\mathrm{out}}^2-r_{\mathrm{in}}^2}\big(T-T_{\mathrm{w}}\big)-\frac{2r_{\mathrm{out}}h_{\mathrm{out}}}{r_{\mathrm{out}}^2-r_{\mathrm{in}}^2}\big(T_{\mathrm{w}}-T_{\mathrm{a}}\big) \tag{S4}$$

linear driving force model

$$\frac{\partial q_{\mathrm{i}}}{\partial t}=k_{\mathrm{i}}(q_{\mathrm{i}}^*-q_{\mathrm{i}}) \tag{S5}$$

pressure drop (Ergun equation)

$$-\frac{\partial P}{\partial z}=\frac{150}{4}\frac{1}{r_{\mathrm{p}}^2}\left(\frac{1-\varepsilon}{\varepsilon}\right)^2\mu v+\frac{1.75}{2}\frac{1}{r_{\mathrm{p}}}\left(\frac{1-\varepsilon}{\varepsilon}\right)\rho|v|v \tag{S6}$$

ideal gas law

$$c_{\mathrm{i}}=\frac{y_{\mathrm{i}}P}{RT} \tag{S7}$$

Table S2: Competitive isotherm models retrieved from Haghpanah et al. [1], Joss et al. [2], and Subraveti et al. [3] for VSA, TSA, and VTSA cases, respectively. $q_i^*$ is the equilibrium solid loading of component $i$.

| Case | Isotherm | Equation | |
|---|---|---|---|
| **VSA** | Dual-site Langmuir | $q_i^* = \frac{q_{\mathrm{sb},i} b_i c_i}{1+\sum_i b_i c_i} + \frac{q_{\mathrm{sd},i} d_i c_i}{1+\sum_i d_i c_i}$ <br> $b_i = b_0 e^{\left(-\frac{\Delta U_{\mathrm{b},i}}{RT}\right)}$ <br> $d_i = d_0 e^{\left(-\frac{\Delta U_{\mathrm{d},i}}{RT}\right)}$ | (S8) |
| **TSA** | Sips | $q_{\mathrm{i}}^* = \frac{q_i^{\infty}(b_i y_i P)^{s_i}}{1+\sum_i (b_i y_i P)^{s_i}}$ <br> $q_i^{\infty} = q_{\mathrm{ref,i}}^{\infty} \exp\left(\chi_i \left(\frac{T}{T_{\mathrm{ref}}} - 1\right)\right)$ <br> $b_i = b_{0,i} \exp\left(\frac{Q_{b,i}}{RT}\right)$ <br> $s_i = s_{\mathrm{ref,i}} + \alpha_i \left(\frac{T_{\mathrm{ref}}}{T} - 1\right)$ | (S9) |
| **VTSA** | Langmuir-Freundlich | $q_i^* = n_{s,i} \frac{(b_i y_i P)^{1/t_i}}{1+\sum_i (b_i y_i P)^{1/t_i}}$ <br> $n_{s,i} = n_{s0} exp\left(\chi_i \left(1 - \frac{T}{T_{\mathrm{ref}}}\right)\right)$ <br> $b_i = b_{0,i} exp\left(\frac{\Delta H_i}{RT_{\mathrm{ref}}} \left(\frac{T_{\mathrm{ref}}}{T} - 1\right)\right)$ <br> $t_i = t_{0,i} + \alpha_i \left(1 - \frac{T_{\mathrm{ref}}}{T}\right)$ | (S10) |

Table S3: Key simulation parameters retrieved from literature for all case studies.

| **Parameter** | **VSA case [1]** | **TSA case [2]** | **VTSA case [3]** |
|---|---|---|---|
| Column length, $L$ (m) | 1.0 | 1.2 | 0.05 |
| Column inner radius, $R_{in}$ (cm) | 14.45 | 1.5 | 1 |
| Column outer radius, $R_{in}$ (cm) | 16.20 | 1.6 | 1.0002 |
| Column voidage, $\epsilon_{\mathrm{B}}$ (-) | 0.37 | 0.35 | 0.25 |
| Particle diameter, $d_{\mathrm{p}}$ (mm) | 1.0 | 2.0 | 1.0 |
| Particle voidage, $\epsilon_{\mathrm{P}}$ (-) | 0.35 | 0.54 | 0.69 |
| Adsorbent density, $\rho_{\mathrm{s}}$ (kg m$^{-3}$) | 1130 | 1085 | 498 |
| Tortuosity, $\tau$ (-) | 3 | - | 3 |
| Wall density, $\rho_{\mathrm{w}}$ (kg m$^{-3}$) | 7800 | 7800 | 7800 |
| Specific heat capacity adsorbent, $C_{\mathrm{p,s}}$ (J kg$^{-1}$ K$^{-1}$) | 1070 | 920 | 1514 |
| Specific heat capacity wall, $C_{\mathrm{p,w}}$ (J kg$^{-1}$ K$^{-1}$) | 502 | 513 | 315 |
| Bed/fluid heat transfer coefficient, $h_{\mathrm{in}}$ (J m$^{-2}$ K$^{-1}$ s$^{-1}$) | 8.6 | 20 | 3 |
| Wall/bed heat transfer coefficient, $h_{\mathrm{out}}$ (J m$^{-2}$ K$^{-1}$ s$^{-1}$) | 2.5 | 100 | 10 |
| Effective gas thermal conductivity, $K_{\mathrm{z}}$ (J m$^{-1}$ K$^{-1}$ s$^{-1}$) | 0.09 | 0.0 | 0.0 |
| Effective wall thermal conductivity, $K_{\mathrm{w}}$ (J m$^{-1}$ K$^{-1}$ s$^{-1}$) | 16 | 0.0 | 0.0 |

# S2 Results

## S2.1 Effect of state variables on the convergence

To identify the key state variables influencing convergence, the component-wise normalized residual norms (scaled 0-1), as defined in Eq. S11, are analyzed over the iterations in Fig. S2. Note that the variables with higher value decay slowly to the convergence.

$$r_n^j = \frac{\mathcal{E}_n^j}{\sum_i \mathcal{E}_n^j} \tag{S11}$$

where $j$ represents state variables, i.e., $y$, $P$, $q$, $T$, and $T_\mathrm{w}$ and $\mathcal{E}_n^j = \|\Delta_{n+1}^j - \Delta_n^j\|_2$, and $n$ is the iterate.

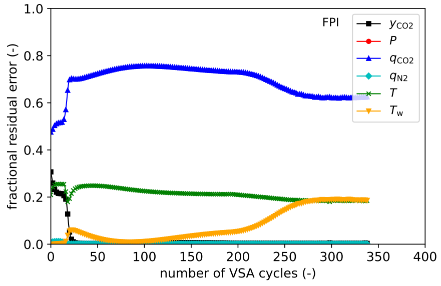


(a)

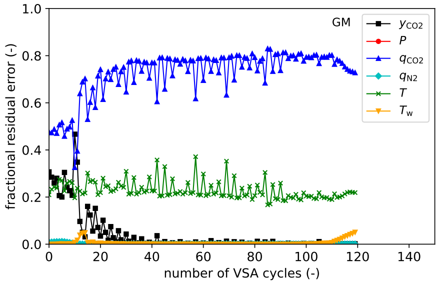


(b)

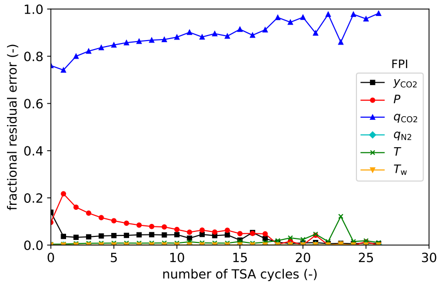


(c)

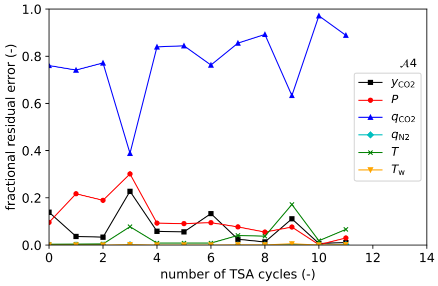


(d)

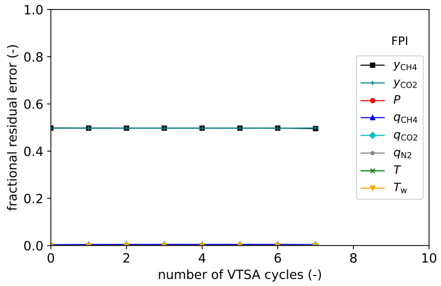


(e)

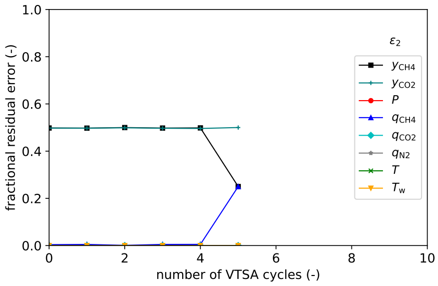


(f)

Figure S1: Fractional contribution of residual for each state variable obtained in CSS convergence of (a)-(b) four-step VSA cycle, (c)-(d) six-step TSA cycle, and (e)-(f) three-step VTSA cycle. Left panel displays without acceleration simulations and the right panel shows with acceleration.

## S2.2 CSS convergence with light product initialization

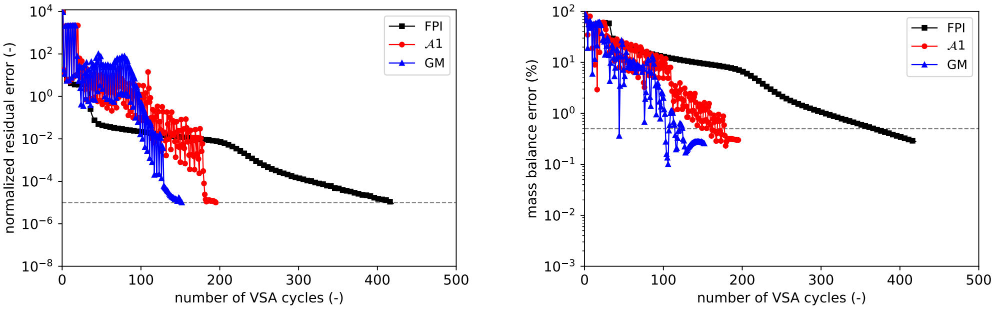


(a) (b)

Figure S2: Convergence of (a) normalized residual and (b) mass balance errors from a light product initial condition to CSS using vector acceleration methods and successive substitution for the four-step VSA cycle. FPI: fixed-point iteration (or successive substitution), ZL: Zienkiewicz-Lohner, and GM: Graves-Morris. The simulations are based on the operating conditions reported in Section 4 of the main paper.

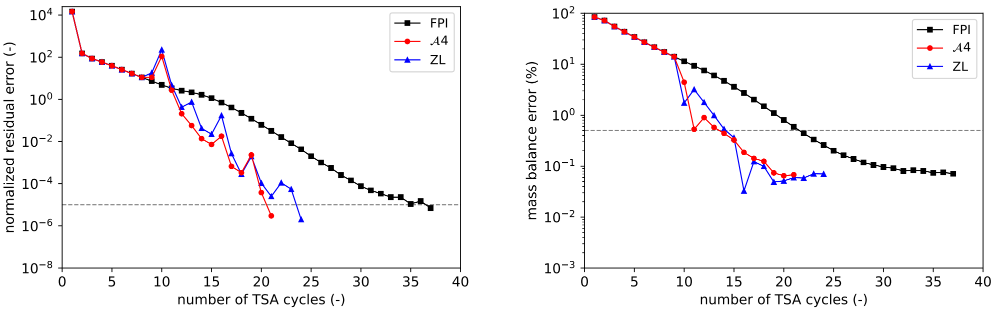


(a) (b)

Figure S3: Convergence of (a) normalized residual and (b) mass balance errors from a light product initial condition to CSS using vector acceleration methods and successive substitution for the six-step TSA cycle. FPI: fixed-point iteration (or successive substitution) and ZL: Zienkiewicz-Lohner. The simulations are based on the operating conditions reported in Section 4 of the main paper.

Table S4: Comparative performance of vector acceleration methods and successive substitution at CSS for the four-step VSA cycle based on the light product initialization and the operating conditions reported in Section 4 of the main paper.

| **method** | **#cycles to CSS** | **comput. time (%)** | **$CO_2$ purity (%)** | **$CO_2$ recovery (%)** |
|---|---|---|---|---|
| **successive substitution** | 420 | 100 | 79.7 | 37.3 |
| $\mathcal{A}$**1** | 195 | 51 | 79.7 | 37.3 |
| **Graves-Morris** | 152 | 41 | 79.7 | 37.3 |

Table S5: Comparative performance of vector acceleration methods and successive substitution at CSS for the six-step TSA cycle based on the light product initialization and the operating conditions reported in Section 4 of the main paper.

| **method** | **#cycles to CSS** | **comput. time (%)** | **$CO_2$ purity (%)** | **$CO_2$ recovery (%)** |
|---|---|---|---|---|
| **successive substitution** | 37 | 100 | 93.1 | 99.3 |
| $\mathcal{A}$**4** | 21 | 51 | 93.1 | 99.3 |
| **Zienkiewicz-Lohner** | 24 | 82 | 93.1 | 99.3 |

## S2.3 Sensitivity analysis

To evaluate the influence of individual operating variables on CSS convergence using vector acceleration methods, a local sensitivity analysis is conducted for the four-step VSA cycle. The improvement in convergence achieved using the Graves-Morris acceleration method is quantified relative to successive substitution using the three performance metrics, i.e., iterations to CSS convergence, computational time, and average logarithmic contraction ratio. Rather than considering these metrics in absolute terms, their ratios between Graves-Morris variant and successive substitution are used to directly assess the relative performance improvement of the acceleration with respect to each operating variable. The performance ratio is defined as:

$$\mathcal{R}^{\pm} = \frac{\text{Performance metric } i \text{ obtained from successive substitution}}{\text{Performance metric } i \text{ obtained from Graves-Morris}} \tag{S12}$$

where $i$ can be iterations to CSS convergence, computational time, and average logarithmic contraction ratio ($\bar{\Lambda}$). The superscript $\pm$ corresponds to either positive or negative perturbation of a parameter with respect to its baseline value. For iterations to CSS convergence and computational time, $\mathcal{R}$ >1 indicates faster convergence and reduction in computational time relative to successive substitution, while for $\bar{\Lambda}$, $\mathcal{R}$ <1 means greater contraction with respect to successive substitution.

And the sensitivity is defined as:

$$\text{Sensitivity} = \frac{\mathcal{R}^{+} - \mathcal{R}^{-}}{2\delta} \tag{S13}$$

where $\delta$ is the perturbation. For this analysis, each operating variable was perturbed $\pm 20\%$ of their baseline value in Section 4, while keeping all other variables constant. The operating variables considered for the sensitivity analysis are: adsorption step duration ($t_{\mathrm{ADS}}$), blowdown step duration ($t_{\mathrm{BLO}}$), evacuation step duration ($t_{\mathrm{EVAC}}$), blowdown step vacuum ($P_{\mathrm{I}}$), evacuation step vacuum ($P_{\mathrm{L}}$), and interstitial feed velocity ($v_0$).

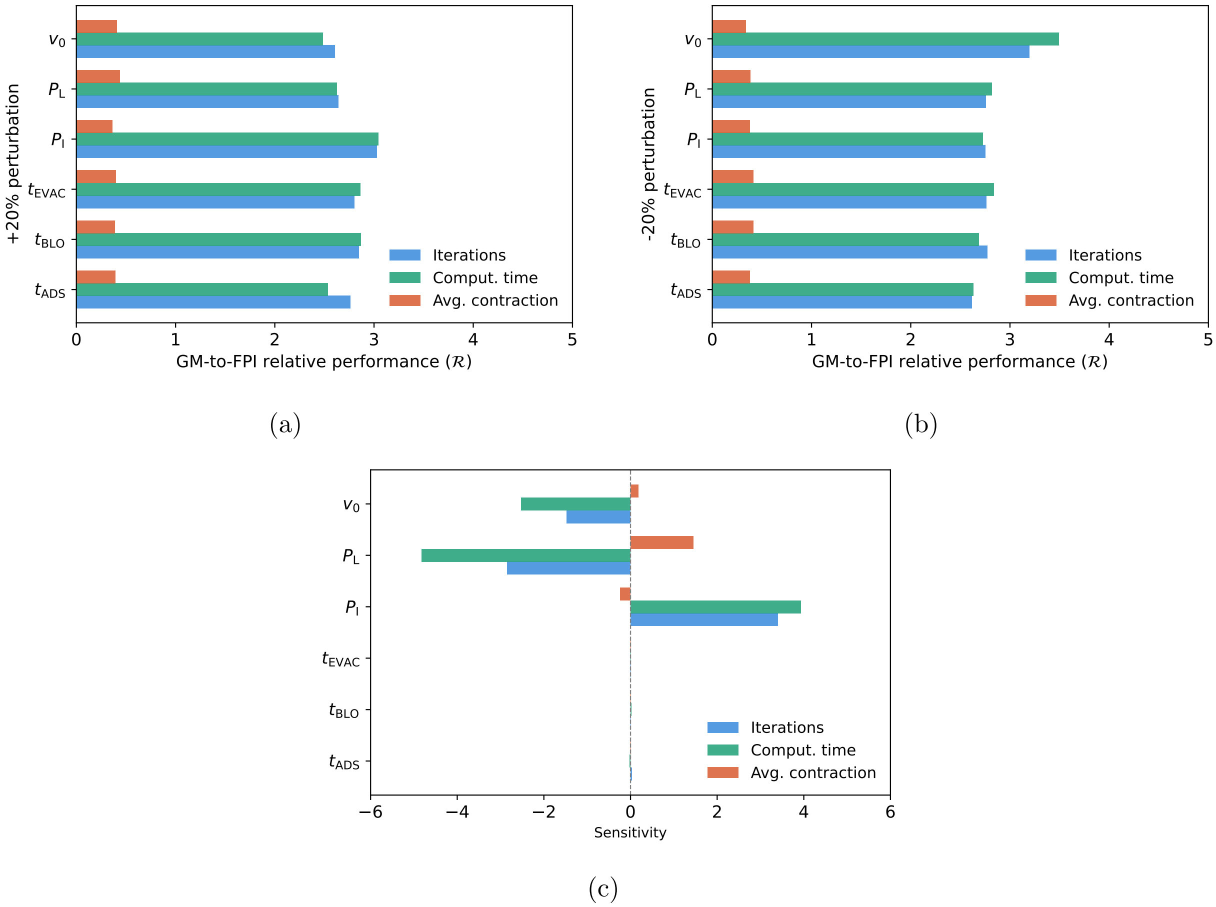


Figure S4: Perturbation sensitivity analysis of the vector acceleration convergence efficiency with respect to six operating parameters. GM-to-FPI performance ratio when each parameter is disturbed by (a) $\delta = +20\%$ ($\mathcal{R}^+$) and (b) $\delta = -20\%$ ($\mathcal{R}^-$) of its nominal value. (c) Sensitivity, indicating the magnitude of each parameter's influence on the ratio of iteration, computational time, and average log contraction between Graves-Morris (GM) and successive substitution (FPI) for the four-step VSA cycle. The simulations are based on the operating conditions reported in Section 4 of the main paper.

## S2.4 Impact of number of fixed-point iterations

Table S6: Impact of number of fixed-point iterations (FPI) in between consecutive accelerations on the convergence speed and computational times for both four-step VSA and six-step TSA cycles.

| | method | #FPI | #cycles to CSS | comput. time (%) | $CO_2$ purity (%) | $CO_2$ recovery (%) |
|---|---|---|---|---|---|---|
| VSA | successive substitution | - | 340 | 100 | 79.7 | 37.2 |
| | $\mathcal{A}1$ | 2 | 340 | 82 | 79.7 | 37.3 |
| | | 3 | 154 | 28 | 79.7 | 37.3 |
| | | 4 | 157 | 49 | 79.7 | 37.3 |
| | | 5 | 165 | 56 | 79.7 | 37.2 |
| | Graves-Morris | 2 | 120 | 40 | 79.7 | 37.2 |
| | | 3 | 121 | 23 | 79.7 | 37.3 |
| | | 4 | 140 | 47 | 79.7 | 37.2 |
| | | 5 | 162 | 52 | 79.7 | 37.2 |
| TSA | successive substitution | - | 28 | 100 | 93.1 | 99.3 |
| | $\mathcal{A}4$ | 2 | 15 | 42 | 93.1 | 99.3 |
| | | 3 | 13 | 40 | 93.1 | 99.3 |
| | | 4 | 19 | 50 | 93.1 | 99.3 |
| | | 5 | 17 | 47 | 93.1 | 99.3 |
| | Zienkiewicz-Lohner | 2 | 23 | 77 | 93.1 | 99.3 |
| | | 3 | 13 | 31 | 93.1 | 99.3 |
| | | 4 | 17 | 44 | 93.1 | 99.3 |
| | | 5 | 15 | 40 | 93.1 | 99.3 |

# References


[1] R. Haghpanah, A. Majumder, R. Nilam, A. Rajendran, S. Farooq, I. A. Karimi, and M. Amanullah. Multiobjective optimization of a four-step adsorption process for postcombustion $CO_2$ capture via finite volume simulation. *Ind. Eng. Chem. Res.*, 52(11):4249–4265, 2013.

[2] L. Joss, M. Gazzani, and M. Mazzotti. Rational design of temperature swing adsorption cycles for post-combustion $CO_2$ capture. *Chem. Eng. Sci.*, 158:381–394, 2017.

[3] S. G. Subraveti and R. Anantharaman. Methane enrichment from dilute sources: Performance limits and implications for methane removal and abatement., 2025. ChemRxiv (pre-print).
